# Real‐World Effectiveness of Osteoporosis Medications in France: A Nationwide Cohort Study

**DOI:** 10.1002/jbm4.10789

**Published:** 2023-07-18

**Authors:** Pauline Bosco‐Lévy, Karine Briot, Nadia Mehsen‐Cetre, James O'Kelly, Gaëlle Désaméricq, Abdelilah Abouelfath, Régis Lassalle, Angela Grelaud, Adeline Grolleau, Patrick Blin, Cécile Droz‐Perroteau

**Affiliations:** ^1^ Bordeaux PharmacoEpi, INSERM CIC‐P 1401 Université de Bordeaux Bordeaux France; ^2^ Service de rhumatologie Hôpital Cochin Paris France; ^3^ Service de rhumatologie CHU Pellegrin‐Tripode Bordeaux France; ^4^ Amgen Ltd Cambridge UK; ^5^ Amgen SAS Boulogne Billancourt France

**Keywords:** ANTIRESORPTIVE MEDICATIONS, FRACTURE INCIDENCE, HEALTHCARE DATABASE, OSTEOPOROSIS

## Abstract

Although drugs for osteoporosis have been demonstrated to be effective in reducing fracture risk in placebo‐controlled clinical trials, data on effectiveness in real‐world practice is limited. Data from the French national health insurance claims database (SNDS) were used to follow five cohorts of women aged ≥55 years after initiating treatment for ≥6 months with either denosumab, zoledronic acid, oral bisphosphonates, raloxifene, or teriparatide in 2014–2016. Fracture incidence was compared within each cohort between the 3 months following initiation (baseline fracture risk) and the 12month, 18month, and 24 month postinitiation periods. Data are presented as incidence rate ratios (IRRs) with their 95% confidence intervals (CIs)s. Overall, 67,046 women were included in the denosumab cohort, 52,914 in the oral bisphosphonate cohort, 41,700 in the zoledronic acid cohort, 11,600 in the raloxifene cohort, and 7510 in the teriparatide cohort. The baseline vertebral fracture rate ranged from 1.74 per 1000 person years (‰PY) in the raloxifene cohort to 34.75‰PY in the teriparatide cohort, and the baseline hip fracture rate from 0.70‰PY in the raloxifene cohort to 10.52‰PY in the zoledronic acid cohort. Compared with the baseline fracture rate, vertebral fractures involving hospitalization were significantly reduced in the 3–24–month postinitiation period with denosumab (IRR 0.6; 95% CI, 0.5–0.7), zoledronic acid (IRR 0.4; 95% CI, 0.3–0.4), teriparatide (IRR 0.3; 95% CI, 0.2–0.5), and oral bisphosphonates (IRR 0.6; 95% CI, 0.4–0.8). Hip fracture incidence was reduced with denosumab (IRR 0.8; 95% CI, 0.6–0.9), but higher for oral bisphosphonates (IRR 1.7; 95% CI, 1.2–2.3); no significant change in hip fracture rate was observed for zoledronic acid, teriparatide, or raloxifene. A reduction in nonvertebral, non‐hip fracture incidence was observed only in the denosumab cohort (IRR 0.8; 95% CI, 0.7–0.9). These findings indicate that treatment with osteoporosis drugs is effective in the real‐world setting. © 2023 The Authors. *JBMR Plus* published by Wiley Periodicals LLC on behalf of American Society for Bone and Mineral Research.

## Graphical abstract

Assessment of the risk fracture in post‐menauposal women in France according to fracture site, osteoporosis treatements and by time periods following treatment initiation, based on a self controlled analysis.

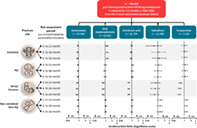

## Introduction

Osteoporosis is a skeletal disease characterized by low bone mass and microarchitectural deterioration of bone tissue, with a consequent increase in bone fragility and susceptibility to fracture.^(^ ^1^ ^)^ Although the diagnosis of the disease relies on the quantitative assessment of bone mineral density, which is a major determinant of bone strength, the clinical significance of osteoporosis lies in the fractures that arise. It has been estimated that approximately one in two postmenopausal women will suffer from an osteoporotic fracture during their remaining lifetime.^(^ ^2^ ^)^ This represents a major health concern, as fragility fractures are known to impair quality of life due to increased disability and more frequent hospital admissions.^(^ ^3^, ^4^ ^)^ Most importantly, osteoporotic fractures are associated with excess mortality.^(^ ^3^, ^4^, ^5^, ^6^, ^7^ ^)^ In France, treatments available for postmenopausal osteoporosis include antiresorptive treatments (bisphosphonates, selective estrogen receptor modulators, and the receptor activator of nuclear factor κB ligand [RANKL] inhibitor denosumab) and an anabolic agent (teriparatide). In placebo‐controlled trials, all approved osteoporosis medications have proven to be effective in reducing the risk of new vertebral fracture in women with postmenopausal osteoporosis. Some of these treatments also have a significant effect on both hip and nonvertebral fractures (namely, alendronate, risedronate, denosumab, and zoledronic acid) and teriparatide has been shown to protect against nonvertebral fractures in general.^(^ ^8^, ^9^ ^)^ Although the anti‐fracture efficacy of osteoporosis treatments has already been demonstrated in selected patient samples in the controlled environment of clinical trials, real‐world data on the effectiveness of these treatments are limited. The objective of this study was to estimate the effectiveness of each osteoporosis treatment by assessing the longitudinal change in fracture incidence in postmenopausal women following initiation of treatment.

## Materials and Methods

### Study design

For this study, data from the French national healthcare claims database, the *Système National des Données de Santé* (SNDS), were used to follow five cohorts of postmenopausal women following initiation of osteoporosis therapy with either oral bisphosphonates (alendronate, risedronate, or ibandronate), zoledronic acid, denosumab, raloxifene, or teriparatide between January 1, 2014 and December 31, 2016. The date of first delivery of a specific osteoporosis treatment was taken as the index date. This was defined as no prior dispensing of the same medication within the previous 12 months (or medication class in the case of oral bisphosphonates). All women were followed for a minimum of 2 years following the index date, or until they died, and information collected on hospitalizations for osteoporotic fractures and any changes in osteoporosis treatment. Their medical and treatment history was extracted from the database for the 3‐year period prior to the index date.

### Ethics

The study was submitted to the French Expert Committee for Research, Studies and Evaluations in the field of Health (CEREES), the appropriate committee for ethical approval of this type of observational study in France, and authorized by the same on May 21, 2019. With regard to data protection, the study was authorized by the French national data protection agency (CNIL) on October 17, 2019 (reference: 919273). Because this was a retrospective study of an anonymized database, ethics committee approval was not required.

### Data source

The SNDS is the comprehensive reimbursement claims database of the French national health insurance system, currently covering around 99% of the total French population.^(^ ^10^, ^11^ ^)^ All beneficiaries are identified by a unique national pseudoanonymized identifier, with which healthcare resource consumption can be tracked over their lifetime. The SNDS compiles data on all reimbursed healthcare consumption in the public and private sectors and in the hospital and community settings. Diagnoses of medical conditions are classified based on the International Classification of Diseases, 10th Revision (ICD‐10). If the beneficiary dies, the cause of death can be accessed through a link to the national deaths register.

### Study population

To limit inclusion to postmenopausal women, only women aged ≥55 were eligible. To be included in the study, they were required to have a look‐back period of 3 years, a follow‐up period of at least 2 years, and at least 6 months of treatment after initiation. Women with a diagnosis of cancer or Paget's disease within the year prior to the index date were excluded, as were women dispensed more than one osteoporosis medication on the index date.

### Exposure

Specific treatments for osteoporosis were identified from the relevant Anatomical Therapeutic Chemical (ATC) code in pharmacy dispensing claims. Treatments of interest included all those approved in this indication in France and used at the appropriate dose for the treatment of osteoporosis. These were oral bisphosphonates (alendronate, risedronate, or ibandronate), zoledronic acid, denosumab, raloxifene, and teriparatide. Hormone replacement therapy was not considered in this study because it was not possible to distinguish clearly between treatment for fracture prevention and symptomatic treatment of menopause.

Because women could sequentially receive multiple treatments, and in order to maximize the size of the cohorts treated with newer therapies, a hierarchical approach was applied to assign the included women to one of five cohorts based on the relative order of entry into the French marketplace of these treatments, with late entry receiving higher priority. Assignment started with the most recently available treatment denosumab, followed by zoledronic acid, teriparatide, oral bisphosphonates, and raloxifene.^(^ ^12^ ^)^ For instance, a woman who initiated oral bisphosphonates within the inclusion period then switched to a more recently marketed treatment such as denosumab at any time within the inclusion period would be in the denosumab cohort with the start of denosumab as the index date.

Treatment discontinuation was defined by the absence of a treatment refill within the 30 days following the time period covered by the last delivery. In this case, the discontinuation date corresponded to the last day covered by the last delivery.

### Outcomes

The primary outcome of interest was the incidence of fragility‐related fractures following the index date. Fragility‐related fractures were identified using a combination of relevant ICD‐10 diagnostic codes listed as the principal, related, or associated diagnosis in the hospital discharge summary and medical procedure codes associated with hospitalizations (Table S1). Incident fractures were categorized into five groups, namely hip fractures, vertebral fractures, wrist/forearm fractures, and non‐hip/nonvertebral fractures (fractures of the wrist/forearm, humerus, clavicle, ribs, pelvis, and leg). These groups are not mutually exclusive (eg, a woman with a wrist fracture will be included both in the “wrist/forearm fracture” group and in the “non‐hip/nonvertebral fracture” group). Sequential occurrence of multiple fracture events at the same body site had to be separated by at least 90 days, without any other fracture identified within this interval of time, in order to consider this new fracture as an independent event.

Incident fractures were assigned to different time periods with respect to the index date. The baseline period covered the first 3 months following the index date, where the treatment benefit on fracture prevention was expected to be minimal.^(^ ^12^ ^)^ Three subsequent risk assessment periods covered the 12, 18, and 24 months following the index date. For the subgroup of women who discontinued their initial treatment, the fracture incidence was also assessed in a postdiscontinuation period covering the period from the date of discontinuation up to the date of reinitiation of an osteoporosis treatment, death, or end of follow‐up, whichever came first.

### Fracture risk factors

A number of variables available in the SNDS were documented for evaluation as covariates of fracture risk. These included age at the index date and comorbidities documented in the 3‐year preindex period (diabetes, endocrine disease, cardiovascular diseases, rheumatoid arthritis, chronic inflammatory bowel disease, lupus erythematosus, and nervous system disorders). These comorbidities were identified from the relevant ICD‐10 codes in the database and used to generate a modified Charlson Comorbidity Index (CCI) score adapted for the SNDS database, as described.^(^ ^13^ ^)^ Any history of fragility‐related fractures was documented. However, information on family history was not available. Bone mineral densitometry, mammography, or colonoscopy performed within the 3‐year preindex period were also documented. However, the results of densitometry are not available in the SNDS so bone density itself is not documented. Medical visits, hospital stays, and use of treatments other than the index medication including bone‐altering medications were also assessed within the year preceding the index date.

### Statistical analysis

#### Principal analysis

The principal analysis consisted of an “as‐treated analysis,” in which women were considered at risk for fracture from the index date until the earliest of the following censure events: treatment discontinuation, switching from index medication to a different study medication, death, or end of follow‐up. Fracture incidence per 1000 person years (‰PY) was computed in each risk assessment period, with the actual time of exposure until the censure event as the denominator. The change in fracture incidence between each risk assessment period was estimated using an own‐control analysis,^(^ ^14^ ^)^ in which all measured and unmeasured individual factors that do not change over time are taken into account, thus minimizing the confounding effect of time‐invariant factors. The fracture incidence in each risk assessment period was compared to that in the 3‐month baseline period. In the subgroup of women who discontinued their index medication, fracture incidence in the postdiscontinuation period was compared to that in the baseline period and to the last on‐treatment risk assessment period. The duration of the postdiscontinuation period was defined as the interval between the date of discontinuation and the end of the follow‐up period, the date of death, or the date of reinitiation of an osteoporosis treatment. The change in fracture incidence was estimated as an incidence rate ratio (IRR) with its 95% confidence interval (95% CI) and the data presented as Forest plots after logarithmic transformation.

#### Stratified subgroup analyses

In order to investigate potential confounding by known risk factors for osteoporotic fractures, the study population was stratified by risk factor and the analysis of IRR by fracture site, treatment cohort, and exposure period estimated by stratum. The risk factors evaluated were age at the index date (four classes: 55–64 years, 65–74 years, 75–79 years, and ≥80 years), history of fractures prior to the index date (four classes: any fracture, hip fracture, vertebral fracture with hospitalization, and non‐hip, nonvertebral fracture), history of specific osteoporosis medication prior to index date (two classes: medication or no medication), prior glucocorticoid use (two classes: ≤3 or >3 prescriptions delivered), dementia at index date (two classes: present or absent), and nervous system disorder at index date (two classes: present or absent).

#### Sensitivity analysis

Two sensitivity analyses were performed in which the definitions of the variables of interest were changed. The first of these used an “intent‐to‐treat” approach in which incidence rate ratios were calculated over the entire period from the index date until the end of the study period (December 31, 2018), or until the woman died, irrespective of whether treatment was discontinued or not. The second analysis was performed to take into account any potential persistent protective effect of treatments beyond the date of discontinuation. In this analysis, an additional “virtual exposure” period was added on to the actual treatment period, set at 365 days for bisphosphonates and at 30 days for the other treatments. Incidence rate ratios were determined for the period from the index date until the end of the “virtual exposure” period (or the date of death or end of follow‐up if either of these events occurred first).

#### Post hoc analysis

In light of unanticipated findings in the oral bisphosphonate cohort, we performed a post hoc analysis in which women who had discontinued their oral bisphosphonate treatment during the 6 months following the index date were included in the analysis.

#### Software

Statistical analysis was performed using Aetion Evidence Platform™, R® (version 3.4.2) and SAS® software (version 9.4) (SAS Institute, Cary, NC, USA).

## Results

### Study population

**Fig. 1:**
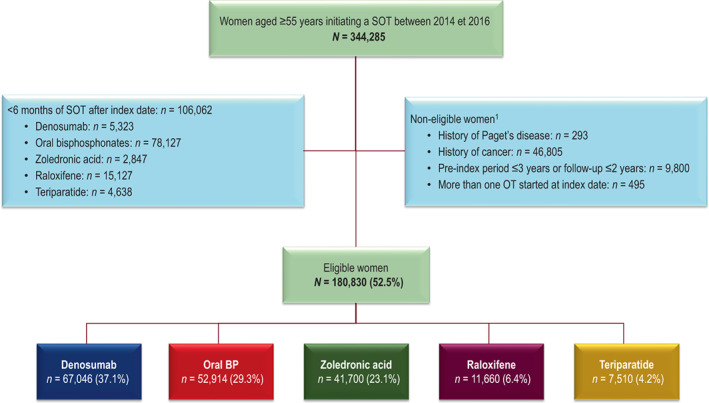
Patient flow diagram. ^1^Women may be ineligible for more than one reason and these categories are thus not mutually exclusive. Percentages are calculated with respect to the previous line. BP = bisphosphonate; SOT = specific osteoporosis treatment.

Overall, 344,285 women aged ≥55 years were documented in the SNDS as having started a specific osteoporosis treatment between 2014 and 2016. Of these, 106,062 (30.8%) were excluded, because they were treated for less than 6 months following the index date. The proportion of women with <6 months treatment varied considerably between treatments, being highest for oral bisphosphonates (73.7% of women with <6 months of treatment) and raloxifene (14.3%). The remaining 180,830 women were eligible for inclusion. Five cohorts were defined using the hierarchical treatment assignment rules and included 67,046 women for the denosumab cohort, 52,914 for the oral bisphosphonate cohort, 41,700 for the zoledronic acid cohort, 11,660 for the raloxifene cohort, and 7510 for the teriparatide cohort. A flow diagram is presented in Fig. 1.

### Characteristics of included women at the index date

The characteristics of the women included at the index date are presented in Table 1. In comparison with the four other cohorts, the raloxifene cohort was younger (mean age of 64 years) at the index date, with a low proportion of women with comorbidities, and the lowest proportion with a history of fracture (5.8%). In contrast, the teriparatide cohort was the oldest (mean age of 76.1 years), with a high proportion of women with comorbidities and the highest proportion with a history of fracture (25.8%). The proportion of women having undergone bone densitometry in the previous 3 years ranged from 61.6% in the teriparatide cohort to 73.6% in the raloxifene cohort. Delivery of an osteoporotic treatment in the previous year was identified in 35.4% of the denosumab cohort, 21.0% of the teriparatide cohort, 18.9% of the zoledronic acid cohort, 10.2% of the oral bisphosphonate cohort, and 10.1% of the raloxifene cohort. For all cohorts with the exception of the oral bisphosphonate cohort, the most frequently delivered prior treatment was an oral bisphosphonate. In addition, around 90% of women in each cohort had received vitamin D or calcium in the previous year.

**Table 1:** Characteristics of Included Women at the Index Date

Characteristic	Denosumab (*n* = 67,046)	Oral BPs (*n* = 52,914)	Zoledronic acid (*n* = 41,700)	Raloxifene (*n* = 11,660)	Teriparatide (*n* = 7510)
Age (years), mean ± SD	74.3 ± 9.2	71.4 ± 9.4	74.1 ± 9.8	63.7 ± 6.7	76.1 ± 9.2
Comorbidities, *n* (%)					
Cardiovascular disease	15,063 (22.5)	9899 (18.7)	11,017 (26.4)	704 (6.0)	2558 (34.1)
Neurological disorders	6958 (10.4)	4992 (9.4)	6056 (14.5)	581 (5.0)	1215 (16.2)
Dementia	1928 (2.9)	1,539 (2.9)	1,933 (4.6)	50 (0.4)	361 (4.8)
Diabetes	4,465 (6.7)	4,081 (7.7)	3,777 (9.1)	394 (3.4)	751 (10.0)
Rheumatoid arthritis	4,383 (6.5)	2,228 (4.2)	3,127 (7.5)	185 (1.6)	563 (7.5)
CCI, mean ± SD	0.3 ± 0.8	0.3 ± 0.7	0.4 ± 0.9	0.1 ± 0.4	0.5 ± 1.0
Fracture history, *n* (%)					
1 fracture	7,191 (10.7)	5,284 (10.0)	6,168 (14.8)	600 (5.1)	1,434 (19.1)
≥2 fractures	2,217 (3.3)	1,238 (2.3)	1,911 (4.6)	77 (0.7)	503 (6.7)
Diagnostic tests					
Bone densitometry (%)	46,660 (69.6)	33,632 (63.6)	26,494 (63.5)	8,576 (73.6)	4,623 (61.6)
Treatment in the year preceding the index date, *n* (%)					
Specific OT	23,737 (35.4)	5,408 (10.2)	7,878 (18.9)	1,180 (10.1)	1,578 (21.0)
Denosumab	0 (0.0)	20 (0.0)	26 (0.1)	≤10	20 (0.3)
Oral BPs	15,506 (23.1)	0 (0.0)	4,312 (10.3)	594 (5.1)	1,141 (15.2)
Zoledronic acid	2,647 (3.9)	151 (0.3)	0 (0.0)	38 (0.3)	111 (1.5)
Raloxifene	2,333 (3.5)	3,619 (6.8)	1,490 (3.6)	0 (0.0)	227 (3.0)
Teriparatide	2831 (4.2)	305 (0.6)	1516 (3.6)	13 (0.1)	0 (0.0)
Corticosteroids	27,917 (41.6)	2,0295 (38.4)	18,163 (43.6)	3,473 (29.8)	3,530 (47.0)
Vitamin D or calcium	62,518 (93.2)	49,088 (92.8)	37,952 (91.0)	10,352 (88.8)	6,936 (92.4)

Abbreviation: BP = bisphosphonates; CCI = Charlson Comorbidity Index; OT = osteoporosis treatment; SD = standard deviation.

### Osteoporosis treatment

The mean follow‐up period ranged from 38 months in the teriparatide cohort to 43 months in the raloxifene cohort (Table 2). Because women were excluded from the study cohort if they received the index treatment for less than 6 months, the median duration of exposure to the index medication was by definition at least 6 months, ranging from 11.8 months in the denosumab cohort to 17.1 months in the teriparatide cohort (Table 2). The proportion of women who discontinued their treatment before the end of the follow‐up period varied from 80.9% in the raloxifene cohort to 92.0% in the teriparatide cohort. The median duration of the postdiscontinuation period ranged from 2 months in the raloxifene cohort to 14 months in the zoledronic acid cohort (Table 2).

**Table 2:** Treatment Exposure

Parameter	Denosumab (*n* = 67,046)	Oral BPs (*n* = 52,914)	Zoledronic acid (*n* = 41,700)	Raloxifene (*n* = 11,660)	Teriparatide (*n* = 7510)
Duration of exposure period (in months)					
Median [IQR]	11.8 [5.9; 24.0]	14.9 [9.1; 26.2]	12.0 [12.0; 23.6]	16.2 [9.5;28.9]	17.1 [11.9;18.4]
Reason for end of exposure, *n* (%)					
Death	585 (0.9)	363 (0.7)	679 (1.6)	23 (0.2)	85 (1.1)
End of follow‐up	7,844 (11.7)	6,600 (12.5)	3,413 (8.2)	1,856 (15.9)	25 (0.3)
Discontinuation	56,978 (85.0)	44,618 (84.3)	35,959 (86.2)	9,437 (80.9)	6,912 (92.0)
Switch	1,639 (2.4)	1,333 (2.5)	1,649 (4.0)	344 (3.0)	488 (6.5)
Duration of postdiscontinuation period (in months)^a^					
Median [IQR]	3.9 [1.6; 18.7]	2.2 [1.3; 10.7]	13.8 [3.3; 26.6]	1.9 [1.2; 5.8]	5.8 [1.3; 21.8]

Abbreviation: IQR = interquartile range; MCR = medication coverage ratio; MPR = medication possession ratio; SD = standard deviation.

^a^ For women who discontinued.

### Fracture incidence

Absolute fracture incidence rates for the different fracture sites during the baseline period are presented in Table 3. The baseline incidence rate for vertebral fractures ranged from 1.74‰PY in the raloxifene cohort to 34.75‰PY in the teriparatide cohort, and the rate for hip fractures from 0.70‰PY in the raloxifene cohort to 10.52‰PY in the zoledronic acid cohort.

**Table 3:** Incidence of Fractures by Site, Treatment, and Exposure Period

Parameter	Denosumab (*n* = 67,046)	Oral BPs (*n* = 52,914)	Zoledronic acid (*n* = 41,700)	Raloxifene (*n* = 11,660)	Teriparatide (*n* = 7510)
Vertebral fracture, (‰PY [95% CI])					
Baseline period	8.78 [7.35–10.21	4.99 [3.77–6.20]	19.22 [16.53–21.90]	1.74 [0.56–4.06]	34.75 [26.23–43.26]
3‐month to 12‐month period	5.72 [4.94–6.49]	2.76 [2.20–3.32]	7.04 [6.10–7.98]	1.06 [0.46–2.09]	14.52 [11.21–17.82]
3‐month to 18‐month period	5.25 [4.62–5.89]	2.77 [2.30–3.25]	7.14 [6.28–8.00]	1.03 [0.42–1.64]	12.03 [9.53–14.53]
3‐month to 24‐month period	4.86 [4.30–5.42]	2.81 [2.38–3.25]	6.86 [6.08–7.64]	0.92 [0.40–1.45]	11.91 [9.48–14.35]
Hip fracture, (‰PY [95% CI])					
Baseline period	8.72 [7.30–10.14]	3.37 2.38–4.37]	10.52 [8.53–12.50]	0.70 [0.08–2.51]	8.65 [4.41–12.89]
3‐month to 12‐month period	7.19 [6.32–8.06]	5.29 [4.51–6.07]	10.65 [9.50–11.80]	0.27 [0.03–0.96]	9.39 [6.73–12.04]
3‐month to 18‐month period	6.61 [5.90–7.32]	5.59 [4.91–6.27]	9.68 [8.69–10.68]	0.84 [0.39–1.60]	9.57 [7.34–11.79]
3‐month to 24‐month period	6.57 [5.92–7.22]	5.58 [4.96–6.20]	9.49 [8.57–10.40]	0.85 [0.35–1.35]	9.68 [7.49–11.87]
Wrist/forearm fracture (‰PY [95% CI])					
Baseline period	8.24 [6.85–9.62]	6.37 [5.00–7.74]	7.20 [5.56–8.84]	4.87 [2.32–7.42]	8.11 [4.00–12.21]
3‐month to 12‐month period	6.68 [5.84–7.51]	6.01 [5.18–6.83]	8.15 [7.14–9.15]	5.32 [3.67–6.96]	10.19 [7.42–12.96]
3‐month to 18‐month period	6.72 [6.00–7.43]	5.94 [5.24–6.64]	7.81 [6.92–8.71]	5.07 [3.72–6.43]	10.00 [7.72–12.27]
3‐month to 24‐month period	6.79 [6.13–7.45]	6.11 [5.46–6.76]	7.60 [6.78–8.42]	4.64 [3.46–5.81]	9.57 [7.39–11.76]
Non‐hip nonvertebral fracture (‰PY [95% CI])					
Baseline period	22.08 [19.81–24.35]	12.13 [10.24–14.02]	21.85 [18.98–24.71]	6.96 [3.91–10.01]	16.23 [10.42–22.04]
3‐month to 12‐month period	18.69 [17.29–20.10]	12.75 [11.54–13.96]	22.74 [21.05–24.43]	10.25 [7.96–12.54]	28.36 [23.73–32.99]
3‐month to 18‐month period	18.29 [17.10–19.48]	13.00 [11.97–14.04]	21.58 [20.09–23.07]	9.79 [7.91–11.67]	25.70 [22.04–29.37]
3‐month to 24‐month period	18.10 [17.02–19.18]	13.28 [12.32–14.23]	21.10 [19.73–22.48]	8.91 [7.28–10.54]	25.28 [21.72–28.83]

Abbreviation: BP = bisphosphonates; CI = confidence interval; PY = person year.

**Fig. 2:**
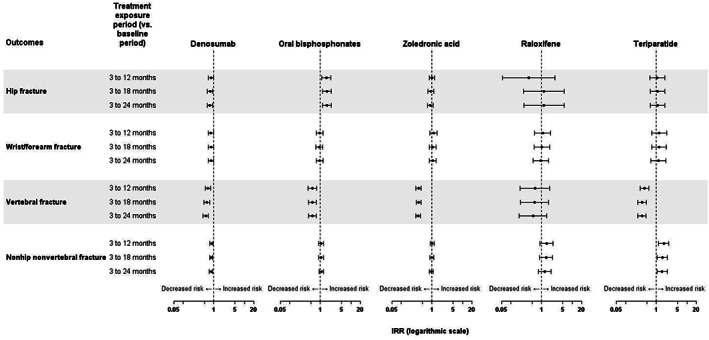
On‐treatment incidence rate ratios for fractures by site and by treatment. The IRR was calculated from the change in fracture incidence between the baseline period and the as‐treated exposure for each treatment exposure period.

Incidence rate ratios over the study periods by fracture site and by treatment are presented in the form of Forest plots in Fig. 2, with full data available in Table S2. During the active exposure periods (3–12 months, 3–18 months, and 3–24 months), the incidence of vertebral fractures decreased compared to the baseline period in all cohorts, with the exception of the raloxifene cohort. For hip fracture, a reduction in incidence was only observed for denosumab for the 3–18–month and 3–24–month periods (Fig. 2). For oral bisphosphonates an increase in fracture rate with respect to baseline was observed in all three exposure periods. (For nonvertebral, non‐hip fractures, a reduction in incidence was observed for denosumab for the 3–24–month period and an increase in incidence for teriparatide for the 3–24–month period). For wrist/forearm fractures, no effects were observed for any treatment.

**Fig. 3:**
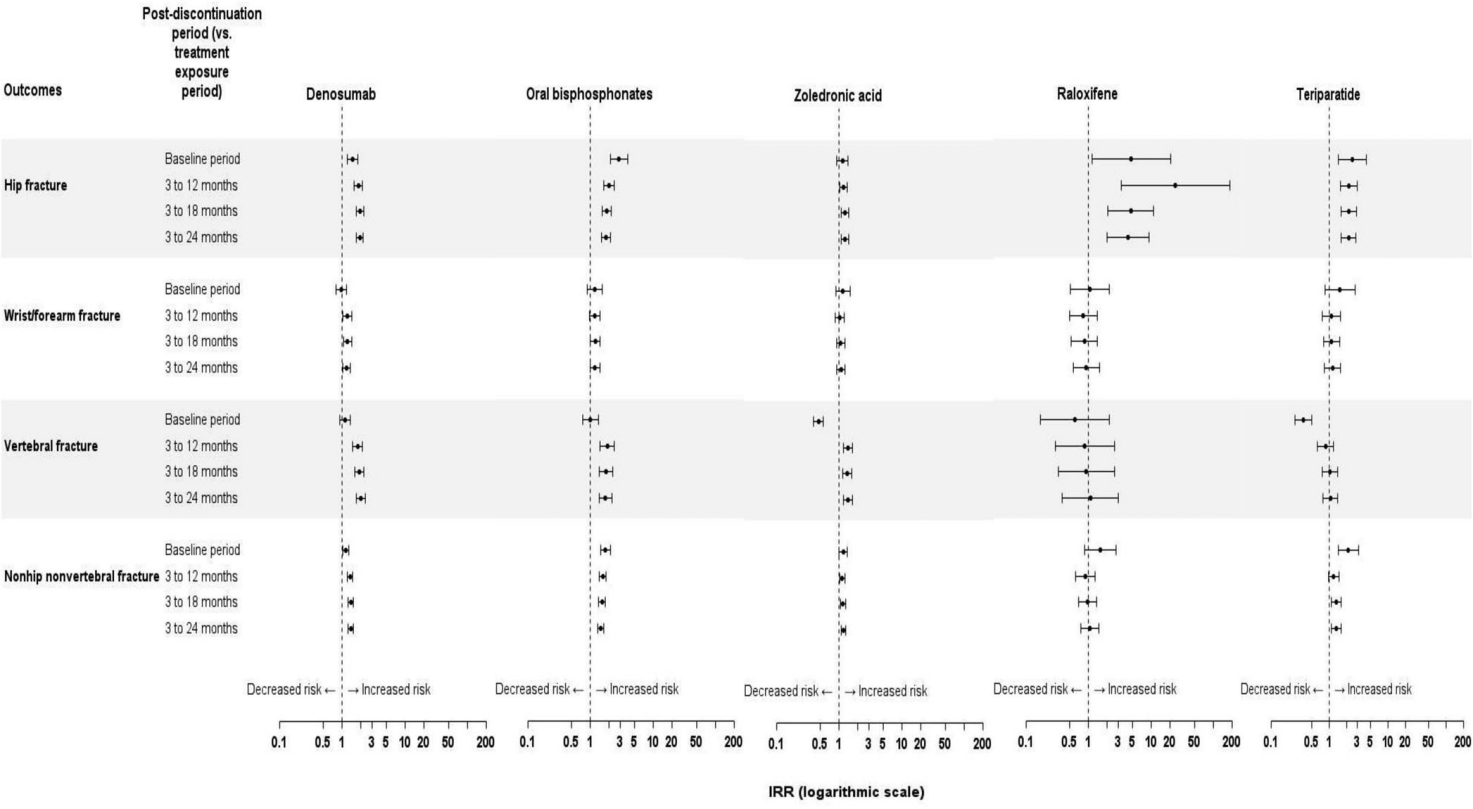
Posttreatment incidence rate ratios for fractures by site and by treatment. The IRR was calculated from the change in fracture incidence between the as‐treated exposure for each treatment exposure period and the postdiscontinuation period.

Following treatment discontinuation, an increase in the incidence of vertebral fracture was observed compared to the previous treatment exposure periods for denosumab (IRR 1.99(95% CI, 1.70–2.32) for the 3–24–month postdiscontinuation period compared to the active exposure period), zoledronic acid (IRR 1.38; 95% CI, 1.17–1.61) and oral bisphosphonate (IRR 1.75; 95% CI, 1.39–2.20) cohorts (Fig. 3). However, no increase in vertebral fracture rate (ie, no “rebound” effect) was observed compared to the baseline period for any of these treatments. For hip fractures and for non‐hip/nonvertebral fractures, an increase in fracture incidence compared to the active exposure period was only observed in the denosumab cohort (Fig. 3). In this cohort, the IRR for the 3–24‐month postdiscontinuation period was 1.91 (95% CI, 1.68–2.18) for hip fractures and 1.36 (95% CI, 1.25–1.49) for non‐hip, nonvertebral fractures.

### Stratified subgroup analyses

Full information on the IRRs for all fracture sites, cohorts, and exposure periods by stratum is provided in Figs. S1–S6. After stratification by age, the decrease in IRR of hip fracture in the zoledronic acid cohort was restricted to the 55–64‐year age class. In the teriparatide cohort, this reduction in IRR was restricted to the 65–74‐year age class for hip fractures and to the 55–64‐year age class for wrist/forearm fractures. An increase in IRR for hip fractures was observed in the ≥80‐year age class of the oral bisphosphonate cohort, as well as for non‐hip/nonvertebral fractures in the 55–64‐year age class of the raloxifene cohort. Moreover, the reduction in the incidence of vertebral fracture observed in the full oral bisphosphonate cohort was not present in the subgroup with a prior fracture.

No major modifications of the IRRs were observed after stratification on prior use of corticosteroids, prior use of specific osteoporosis medication, history of dementia, and history of nervous system disorders for any of the cohorts.

### Sensitivity analyses

The sensitivity analysis yielded similar results to those of the principal analysis, for all cohorts and all fracture sites considered (see Tables S4 and S5).

### Post hoc analysis

A post hoc analysis was conducted in the 78,127 women who were not initially included in the oral bisphosphonate cohort because they discontinued their treatment within the first 6 months following the index date. In these women, the vertebral fracture incidence rate in the baseline period was much higher than in those treated for >6 months (12.1 versus 5.0‰PY), suggesting that this group of women was at much higher fracture risk. However, an increase in fracture rate was still observed in the 3–6‐month period, with an incidence risk ratio similar to that observed in the principal analysis (IRR 1.51; 95% CI, 1.13–2.04).

## Discussion

The objective of this study was to determine the change in incidence of low‐trauma fractures following initiation of a specific osteoporosis treatment in a real‐world population of women aged ≥55 years. Compared to the baseline period, a reduction in the incidence of vertebral fractures in the posttreatment periods was observed for women treated with denosumab, zoledronic acid, teriparatide, and oral bisphosphonates. In addition, a reduction in the incidence of hip and non‐hip/nonvertebral fractures was also observed in women receiving denosumab. No benefits on the incidence of any fracture types were observed for women receiving raloxifene. The incidence of all types of fractures increased again after treatment discontinuation.

Women aged ≥55 years were assigned to one of five cohorts on the basis of the most recently marketed osteoporosis treatment, with the largest cohorts being women treated with denosumab, zoledronic acid, and oral bisphosphonates. Their characteristics differed to some extent between the five cohorts. In particular, women receiving raloxifene were generally younger whereas those prescribed teriparatide were typically older with more comorbidities. For this reason, the intrinsic fracture risk in each cohort is different, and this is reflected in the proportion of women with a history of prior fracture and the fracture incidence rate during the baseline period. Between 10% and 35% of women had already received a prior osteoporosis treatment at the index date. This was to be expected, as the index date was the first prescription of the most recently introduced treatment, with certain women having been treated with other medications in the previous year.

The method used in this study allows comparisons within a population rather than between populations and thus minimizes the risk of bias due to patient‐specific confounding factors.^(^ ^14^, ^15^ ^)^ This approach is useful because it allows for the comparison of groups with different characteristics by using each woman as her own control, rather than attempting comparisons between groups. This minimizes the risk of confounding by important risk factors for osteoporotic fracture that are not documented in the SNDS, such as a family history of osteoporotic fracture and bone density. In this approach, a baseline period of 3 months following treatment initiation is taken to be the reference period for determining the rate ratio.^(^ ^15^ ^)^ The reason for this choice, rather than using the period immediately preceding treatment as the reference, is that osteoporosis treatments are frequently initiated as a consequence of occurrence of a fracture, and that using the pretreatment period as the reference may lead to an overestimate of baseline fracture incidence and thus of confusing possible treatment effects with regression to the mean of the fracture rate. The choice of the first 3 months as the reference is justified by the fact that the pharmacological effect of bisphosphonates, teriparatide, and raloxifene is not immediate. For these treatments, significant changes in bone mineral density, fracture reductions, or biomarkers have not been noted earlier than 6–12 months of therapy.^(^ ^16^, ^17^, ^18^ ^)^ The choice of the baseline period duration may, however, not be appropriate for denosumab, for which a reduction in bone‐resorption has been observed since as early as the first month of treatment.^(^ ^19^ ^)^ For this reason, our approach may underestimate the real positive effect of denosumab on fracture risk. The same approach has also been previously employed in effectiveness studies of other therapies^(^ ^20^, ^21^ ^)^ (as well as of the impact of osteoporosis treatment on fracture incidence in other treatment settings). The earliest study in osteoporosis, by Abelson and colleagues,^(^ ^15^ ^)^ evaluated hip fracture incidence after initiation of oral bisphosphonate treatment in >200,000 postmenopausal women in a US insurance claims database and demonstrated a significant reduction in incidence rate during the on‐treatment period compared to the baseline. Since then, a number of other studies have used this approach to demonstrate a reduction in fracture incidence at various sites following initiation of a specific osteoporosis treatment.^(^ ^12^, ^14^, ^22^, ^23^, ^24^ ^)^

Our findings in the real‐world treatment setting are not entirely consistent with those from the pivotal randomized clinical trials of these treatments. For denosumab, we observed a significant reduction in the incidence of hip, vertebral, and non‐hip/nonvertebral fractures, which is consistent with the clinical trial data.^(^ ^25^ ^)^ In contrast, we only observed a protective effect of zoledronic acid or teriparatide against vertebral fractures, whereas clinical trials have described a reduction in the incidence of nonvertebral fractures with both these agents^(^ ^26^, ^27^ ^)^ and a reduction in the incidence of hip fracture with zoledronic acid.^(^ ^26^ ^)^ However, it should be noted that the treatment duration with osteoporotic treatments in the present real‐world study was shorter than the 2 years (teriparatide) or 3 years (zoledronic acid) of exposure implemented in the clinical trials.

The principal unexpected finding from this study, which is in contradiction with most data from previous interventional^(^ ^28^, ^29^, ^30^ ^)^ and observational studies,^(^ ^15^, ^22^ ^)^ was that the IRR for hip fracture in women treated with oral bisphosphonates increased in the different on‐treatment periods in comparison with the initial 3‐month baseline period. The codes related to hip fractures were closely checked and no excess of atypical femoral fractures, which may be facilitated by bisphosphonates,^(^ ^31^ ^)^ were observed. We have no explanation for this paradoxical finding, other than it may be an artifact due to the presence of some unmeasured time‐dependent confounding factor that is specific to the cohort treated with oral bisphosphonates. It should, however, be noted that, using a different approach (an exposed and unexposed cohort compared with a marginal structural Cox model), another study in a subset of the SNDS database failed to observe a reduction in hip fracture rate following initiation of an oral bisphosphonate (although an increase was not seen).^(^ ^32^ ^)^ The authors suggested that the absence of effect may have been due to the fact that treatment exposure was too short (12 or 17 months according to the definition) to be effective. Similarly, in the own‐control analysis performed in a US health insurance claims database, which used a methodology very similar to our own, no effect on hip fracture rate was observed in the 12 months following initiation of an oral bisphosphonate.^(^ ^12^ ^)^ One explanation could be that our analysis does not include patients with <6 months of treatment with oral bisphosphonates (OBs), who represent a high proportion of OB users. These earlier discontinuers appear to be at a higher fracture risk in the baseline period than those who continue their OB treatment for >6 months (the incidence of hip fracture in the early period was 12.4‰PY in early discontinuers and 3.4‰PY in OB treatment persistent patients). By excluding these high‐risk patients, we may have masked the potential beneficial effect of OB on fracture reduction.

Another finding of this study was that the fracture incidence rate increased after discontinuation of treatment. This was observed for the majority of treatments and all fracture sites, with the exception of vertebral fractures. However, the increase in fracture incidence after discontinuation of zoledronic acid was less marked than that observed with denosumab or teriparatide. Similar findings have been reported in another observational study from the United States, which found that the risk of fracture increased after treatment discontinuation in women receiving oral bisphosphonates but not in those receiving zoledronic acid.^(^ ^33^ ^)^ Given that 80% to 92% of women discontinued their treatment within 2 years of initiation, a large proportion of women are exposed to an avoidable fracture risk at any one time. It is thus important that physicians encourage women to remain on their osteoporosis treatment over the long term, or at least to switch to another active treatment if the current one is poorly tolerated.^(^ ^34^ ^)^ Because the benefit from osteoporosis treatments is not tangible (avoidance of a fracture) and the absolute fracture rate is rather low, some women taking these treatments may consider that they are not useful,^(^ ^35^ ^)^ and this perception needs to be challenged.

The fracture rates in treated patients reported in the present study may be compared with the fracture incidence rate in the French general population as a whole (all individuals aged ≥50 years, both men and women), that has recently been determined using the same data source (the SNDS).^(^ ^7^ ^)^ In this study, only 17% of patients had been treated with a specific osteoporosis treatment at the time of fracture. The crude annual incidence of fracture was 2.2‰ for hip fractures and 0.3‰ for vertebral fractures.^(^ ^7^ ^)^ Incidence rates in this predominantly untreated population are lower than in those estimated in the present study, which can be explained first by the fact that the previous study also included men, who are at lower risk for fracture than women and second because the women in the present study were, by definition, all treated and thus presumably had identified risk factors for osteoporotic fracture that had incited their treating physicians to prescribe anti‐osteoporotic treatment.

The strengths of the study include the large numbers of women included, which correspond to all eligible women treated with a reimbursed specific osteoporosis treatment for over 6 months. This was made possible by extracting data from the SNDS, which contains exhaustive information on all reimbursed healthcare consumption in France. Second, the self‐controlled analysis allowed the effect of patient‐related confounding factors to be minimized. This study has some limitations. First, we cannot rule out the possibility of a residual confounding bias by indication that is inherent to the study design. Osteoporosis medications are mostly prescribed following a fracture and, by including patients at treatment initiation, we may have selected more severe patients. However, we have attempted to address this bias by performing a sensitivity analysis stratified on fracture history, which did not qualitatively change the findings. In addition, the study population was limited to patients with at least 6 months of treatment after initiation. This choice was made to ensure that enough patients could be exposed to osteoporosis in the risk assessment periods and thus evaluable. The group of patients who were the most impacted by this criterion was the oral bisphosphonates users, which was not surprising because some of the other osteoporosis treatments are given on a semiannual or annual frequency. The high proportion of oral bisphosphonate users who discontinue their treatment within the first 6 months after treatment initiation, are in line with the suboptimal compliance and persistence reported by previous studies in these patients.^(^ ^36^ ^)^ By applying this inclusion criterion, results of the present study can only be extrapolated to patients with at least 6 months of treatment use. Another limitation is related to the methodological approach applied. If the method allows to account for the effect of individual time‐invariant confounders, it fails to take into account patient‐related variables that vary over time, such as age. We managed to address this limitation by performing stratified analyses on age, which highlighted some variation in IRR between age strata for certain fracture types and cohorts, but with no consistent pattern having emerged. Moreover, no obvious differences were found in IRRs for fractures with different exposure periods, and the maximum follow‐up period did not exceed 24 months, so the age‐related increase in fracture risk is expected to be minimal. In addition, analysis conducted in the German data using the same approach as the present study, showed no major impact of increased age on fracture risk.^(^ ^24^ ^)^ Certain limitations of the study are inherent to the SNDS database. For example, information on the results of bone densitometry or radiography are not available and it is not possible to know whether the medications delivered were actually taken. Information on menopausal status is not available, so age ≥55 years was used as a proxy selection criterion for this. In addition, it should be noted that many important fracture risk factors cannot be documented in the SNDS, including bone mineral density (*T*‐score), low body weight, falls, and lifestyle factors. The same is true for potential protective factors such as dietary supplementation with over‐the‐counter calcium or vitamin D preparations (although it should be noted that use of such supplementation in the target population in France is very low^(^ ^37^ ^)^). The SNDS does not provide any information on the reasons for choosing to initiate, switch, or discontinue a given treatment, and these reasons may also have an impact on fracture risk. In the present study, identification of fractures was based on hospitalization diagnosis codes and not on imaging data (as is usually the case in randomized clinical trials), and this may have induced a nondifferential misclassification bias. In particular, the incidence of vertebral and wrist fractures may be underestimated, because these do not necessarily require hospitalization and only fractures that led to hospitalization could be documented. Another limitation is that not all included women were treatment‐naïve. However, a stratified analysis comparing treatment‐naïve and previously treated women failed to reveal any influence of this variable on the impact of the index treatment. Finally, although patients were selected from the SNDS on the basis of diagnostic codes for fragility fractures, it cannot be excluded that certain fractures in the study population were not related to osteoporosis, but such fractures should be very rare.

In conclusion, this study reported a fracture risk reduction of zoledronic acid, teriparatide, and oral bisphosphonates on vertebral fractures and of denosumab on vertebral, peripheral, and hip fractures among postmenopausal women treated for at least 6 months.

## Author Contributions

**Pauline Bosco‐Lévy:** Conceptualization; investigation; methodology; validation; writing – original draft; writing – review and editing. **Karine Briot:** Conceptualization; investigation; validation; writing – review and editing. **Nadia Mehsen‐Cetre:** Conceptualization; investigation; validation; writing – review and editing. **James O'Kelly:** Conceptualization; investigation; validation; writing – review and editing. **Gaëlle Désaméricq:** Conceptualization; investigation; validation; writing – review and editing. **Abdelilah Abouelfath:** Conceptualization; investigation; methodology; project administration; validation; visualization; writing – review and editing. **Régis Lassalle:** Conceptualization; data curation; formal analysis; investigation; methodology; software; validation; writing – review and editing. **Angela Grelaud:** Conceptualization; investigation; validation; writing – review and editing. **Adeline Grolleau:** Investigation; validation; writing – review and editing. **Patrick Blin:** Conceptualization; investigation; methodology; writing – review and editing. **Cécile Droz‐Perroteau:** Funding acquisition; supervision.

## Disclosures

PBL, RL, AA, AG, PB, and CDP are researchers at Bordeaux PharmacoEpi, a research platform of the University of Bordeaux and its subsidiary the ADERA, which performs financially supported studies for public and private partners, in compliance with the ENCePP Code of Conduct. JO'K and GD are employees of Amgen and hold stock ownership in the company. NMC has no conflict of interest to disclose. KB has received honoraria for lectures, presentations, speakers' bureaus, manuscript writing or educational events from Amgen, Theramex and Lilly.

### Peer Review

The peer review history for this article is available at https://www.webofscience.com/api/gateway/wos/peer‐review/10.1002/jbm4.10789.

## Supporting information


**Data S1.** Supporting Information.
Tables S1–S5.
Figs. S1–S6.

## Data Availability

Access to data in the SNDS is available exclusively to institutions who meet the criteria for access to confidential data, following procurement of consent from the Ethic and Scientific Committee (CESRESS) of the Health Data Hub (HDH) and from the French data protection authority (CNIL). The contact address for the INDS is *Institut National des Données de Santé* (INDS), 19 rue Arthur Croquette, 94220 Charenton‐le‐Pont,; E‐mail: contact@indsante.fr; Website: https://www.indsante.fr/fr.
