# Supplementary material for: Real‐World Effectiveness of Osteoporosis Medications in France: A Nationwide Cohort Study
Source: JBMR Plus. 2023 Jul 18;7(9):e10789. doi: 10.1002/jbm4.10789 (PMC10494501; doi:10.1002/jbm4.10789)
Supplement: Supplementary file 1 — Data S1. Supporting Information. Tables S1–S5. Figs. S1–S6. [file JBM4-7-e10789-s001.docx]

**Real-World Effectiveness of Osteoporosis Medications in France:
A Nationwide Cohort Study**

**Supplemental Material**

**Supplemental Table 1.** Codes list of fractures outcomes

| **Outcome** | **ICD-10 codes** |
| --- | --- |
| Hip  (excluding the complications of hip replacement) | S72.0, S72.1, S72.2, S32.4, M80.05,  *These codes must not be associated during the same stay with T84^1^ or Y83^2^ diagnostic codes or NEEA002/NEKA001-9 procedure codes^3^* |
| Wrist/forearm | S52, S62.0, S62.1, S62.2, S62.3, S62.4, M80.03 |
| Vertebral fracture | S22.0, S22.1, and S32 (excluding S32.3-5 and S32.8), M80.08, T08 |
| Nonvertebral  (hip, wrist/forearm, humerus, clavicle, ribs, pelvis, and leg) | S72.0, S72.1, S72.2, S32.4, M80.05  *These codes must not be associated during the same stay with T84^1^ or Y83^2^ diagnostic codes or NEEA002/NEKA001-9 procedure codes^3^*  S22.3, S22.4, S22.5, S32.3, S32.4, S32.5, S32.8, S42.0, S42.1, S42.2, S42.3, S42.4, S52, S62.0, S62.1, S62.2, S62.3, S62.4, S72.3, S72.4, S72.5, S72.6, S72.7, S72.8, S72.9, S82, S92.0, S92.1, S92.2, S92.3, M80.01, M80.02, M80.03 |
| Non-hip, nonvertebral  (wrist/forearm, humerus, clavicle, ribs, pelvis, and leg) | S22.3, S22.4, S22.5, S32.3, S32.4, S32.5, S32.8, S42.0, S42.1, S42.2, S42.3, S42.4, S52, S62.0, S62.1, S62.2, S62.3, S62.4, S72.3, S72.4, S72.5, S72.6, S72.7, S72.8, S72.9, S82, S92.0, S92.1, S92.2, S92.3, M80.01, M80.02, M80.03 |
| ^1^Complications of internal orthopaedic prosthetic devices, implants and grafts  ^2^Surgical operation and other surgical procedures as the cause of abnormal reaction of the patient, or of later complication, without mention of misadventure at the time of the procedure  ^3^NEEA002: reduction of a prosthesis dislocation of the coxofemoral joint, by arthrotomy; NEKA001-NEKA009: change of acetabular and/or femoral parts of a total hip replacement | |

Supplemental Table 2. Incidence rate ratios by site, treatment and exposure period

|  | **Denosumab N = 67,046** | **Oral BPs N = 52,914** | **Zoledronic acid N = 41,700** | **Raloxifene N = 11,660** | **Teriparatide N = 7,510** |
| --- | --- | --- | --- | --- | --- |
| **Vertebral fracture, IRR [95% CI])** | | | |  |  |
| Baseline period | 1 | 1 | 1 | 1 | 1 |
| 3- to 12-month period | 0.65 [0.53; 0.81] | 0.55 [0.40; 0.76] | 0.37 [0.30; 0.44] | 0.61 [0.20; 1.86] | 0.42 [0.30; 0.58] |
| 3- to 18-month period | 0.60 [0.49; 0.73] | 0.56 [0.41; 0.75] | 0.37 [0.31; 0.45] | 0.59 [0.21; 1.70] | 0.35 [0.25; 0.48] |
| 3- to 24-month period | 0.55 [0.45; 0.68] | 0.56 [0.42; 0.75] | 0.36 [0.30; 0.43] | 0.53 [0.19; 1.50] | 0.34 [0.25; 0.47] |
| **Hip fracture, IRR [95% CI])** | | | | | |
| Baseline period | 1 | 1 | 1 | 1 | 1 |
| 3- to 12-month period | 0.82 [0.67; 1.01] | 1.57 [1.13; 2.18] | 1.01 [0.81; 1.26] | 0.39 [0.05; 2.74] | 1.09 [0.62; 1.91] |
| 3- to 18-month period | 0.76 [0.62; 0.92] | 1.66 [1.21; 2.28] | 0.92 [0.74; 1.14] | 1.20 [0.26; 5.55] | 1.11 [0.64; 1.90] |
| 3- to 24-month period | 0.75 [0.62; 0.91] | 1.66 [1.21; 2.27] | 0.90 [0.73; 1.11] | 1.21 [1.27; 5.48] | 1.12 [0.65; 1.92] |
| **Wrist/forearm fracture IRR [95% CI])** | | | | | |
| Baseline period | 1 | 1 | 1 | 1 | 1 |
| 3- to 12-month period | 0.81 [0.66; 1.00] | 0.94 [0.73; 1.22] | 1.13 [0.87; 1.47] | 1.09 [0.59; 2.01] | 1. 26 [0.71; 2.23] |
| 3- to 18-month period | 0.82 [0.67; 1.00] | 0.93 [0.73; 1.19] | 1.08 [0.84; 1.40] | 1.04 [0.58; 1.87] | 1.23 [0.71; 2.15] |
| 3- to 24-month period | 0.82 [0.68; 1.00] | 0.96 [0.75; 1.22] | 1.06 [0.82; 1.36] | 0.95 [0.53; 1.70] | 1.18 [0.68; 2.06] |
| **Non-hip non-vertebral fracture (IRR [95% CI])** | | | | | |
| Baseline period | 1 | 1 | 1 | 1 | 1 |
| 3- to 12-month period | 0.85 [0.75; 0.96] | 1.06 [0.88; 1.26] | 1.04 [0.90; 1.21] | 1.47 [0.90; 2.41] | 1. 75 [1.18; 2.59] |
| 3- to 18-month period | 0.83 [0.73; 0.94] | 1.07 [0.90; 1.28] | 0.99 [0.85; 1.15] | 1.41 [0.87; 2.27] | 1.58 [1.08; 2.33] |
| 3- to 24-month period | 0.82 [0.73; 0.92] | 1.09 [0.92; 1.30] | 0.97 [0.83; 1.12] | 1.28 [0.80; 2.06] | 1.56 [1.06; 2.29] |

BP: bisphosphonates; CI: confidence interval; IRR: incidence rate ratio.

Supplemental Table 3. Incidence rate ratios by site, treatment and exposure period

|  | **Denosumab N = 67,046** | **Oral BPs N = 52,914** | **Zoledronic acid N = 41,700** | **Raloxifene N = 11,660** | **Teriparatide N = 7,510** |
| --- | --- | --- | --- | --- | --- |
| **Vertebral fracture, IRR [95% CI])** | | | |  |  |
| Baseline period | 1 | 1 | 1 | 1 | 1 |
| 3- to 12-month period | 0.65 [0.53; 0.81] | 0.55 [0.40; 0.76] | 0.37 [0.30; 0.44] | 0.61 [0.20; 1.86] | 0.42 [0.30; 0.58] |
| 3- to 18-month period | 0.60 [0.49; 0.73] | 0.56 [0.41; 0.75] | 0.37 [0.31; 0.45] | 0.59 [0.21; 1.70] | 0.35 [0.25; 0.48] |
| 3- to 24-month period | 0.55 [0.45; 0.68] | 0.56 [0.42; 0.75] | 0.36 [0.30; 0.43] | 0.53 [0.19; 1.50] | 0.34 [0.25; 0.47] |
| **Hip fracture, IRR [95% CI])** | | | | | |
| Baseline period | 1 | 1 | 1 | 1 | 1 |
| 3- to 12-month period | 0.82 [0.67; 1.01] | 1.57 [1.13; 2.18] | 1.01 [0.81; 1.26] | 0.39 [0.05; 2.74] | 1.09 [0.62; 1.91] |
| 3- to 18-month period | 0.76 [0.62; 0.92] | 1.66 [1.21; 2.28] | 0.92 [0.74; 1.14] | 1.20 [0.26; 5.55] | 1.11 [0.64; 1.90] |
| 3- to 24-month period | 0.75 [0.62; 0.91] | 1.66 [1.21; 2.27] | 0.90 [0.73; 1.11] | 1.21 [1.27; 5.48] | 1.12 [0.65; 1.92] |
| **Wrist/forearm fracture IRR [95% CI])** | | | | | |
| Baseline period | 1 | 1 | 1 | 1 | 1 |
| 3- to 12-month period | 0.81 [0.66; 1.00] | 0.94 [0.73; 1.22] | 1.13 [0.87; 1.47] | 1.09 [0.59; 2.01] | 1. 26 [0.71; 2.23] |
| 3- to 18-month period | 0.82 [0.67; 1.00] | 0.93 [0.73; 1.19] | 1.08 [0.84; 1.40] | 1.04 [0.58; 1.87] | 1.23 [0.71; 2.15] |
| 3- to 24-month period | 0.82 [0.68; 1.00] | 0.96 [0.75; 1.22] | 1.06 [0.82; 1.36] | 0.95 [0.53; 1.70] | 1.18 [0.68; 2.06] |
| **Non-hip non-vertebral fracture (IRR [95% CI])** | | | | | |
| Baseline period | 1 | 1 | 1 | 1 | 1 |
| 3- to 12-month period | 0.85 [0.75; 0.96] | 1.06 [0.88; 1.26] | 1.04 [0.90; 1.21] | 1.47 [0.90; 2.41] | 1. 75 [1.18; 2.59] |
| 3- to 18-month period | 0.83 [0.73; 0.94] | 1.07 [0.90; 1.28] | 0.99 [0.85; 1.15] | 1.41 [0.87; 2.27] | 1.58 [1.08; 2.33] |
| 3- to 24-month period | 0.82 [0.73; 0.92] | 1.09 [0.92; 1.30] | 0.97 [0.83; 1.12] | 1.28 [0.80; 2.06] | 1.56 [1.06; 2.29] |

BP: bisphosphonates; CI: confidence interval; IRR: incidence rate ratio.

Supplemental Table 4. Incidence rate ratios by site, treatment and exposure period in intent to treat analysis (sensitive analysis 1)

|  | **Denosumab N = 67,046** | **Oral BPs N = 52,914** | **Zoledronic acid N = 41,700** | **Raloxifene N = 11,660** | **Teriparatide N = 7,510** |
| --- | --- | --- | --- | --- | --- |
| **Vertebral fracture, IRR [95% CI])** | | | |  |  |
| Baseline period | 1 | 1 | 1 | 1 | 1 |
| 3- to 12-month period | 0.73 [0.60; 0.89] | 0.62  [0.46; 0.83] | 0.37  [0.31; 0.45] | 0.59  [0.20; 1.77] | 0.40  [0.29; 0.56] |
| 3- to 18-month period | 0.74 [0.62; 0.89] | 0.70  [0.53; 0.92] | 0.39  [0.33; 0.46] | 0.67  [0.25; 1.81] | 0.34  [0.25; 0.47] |
| 3- to 24-month period | 0.75 [0.63; 0.90] | 0.72  [0.55; 0.93] | 0.38  [0.33; 0.45] | 0.59  [0.22; 1.57] | 0.32  [0.24; 0.43] |
| **Hip fracture, IRR [95% CI])** | | | | | |
| Baseline period | 1 | 1 | 1 | 1 | 1 |
| 3- to 12-month period | 0.92 [0.76; 1.11] | 1.75  [1.27; 2.41] | 1.01 [0.81; 1.25] | 0.81  [0.16; 4.20] | 1.28 [0.74; 2.21] |
| 3- to 18-month period | 0.89 [0.75; 1.07] | 1.88  [1.38; 2.57] | 0.96 [0.78; 1.18] | 1.57  [0.36; 6.83] | 1.41 [0.84; 2.38] |
| 3- to 24-month period | 0.91 [0.76; 1.09] | 1.94  [1.43; 2.63] | 0.97 [0.80; 1.19] | 2.03  [0.48; 8.50] | 1.48 [0.89; 2.48] |
| **Wrist/forearm fracture IRR [95% CI])** | | | | | |
| Baseline period | 1 | 1 | 1 | 1 | 1 |
| 3- to 12-month period | 0.87 [0.71; 1.06] | 0.96  [0.75; 1.23] | 1.14  [0.88; 1.48] | 1.05  [0.58; 1.92] | 1.32  [0.75; 2.32] |
| 3- to 18-month period | 0.87  [0.72; 1.04] | 0.98  [0.77; 1.24] | 1.11  [0.87; 1.42] | 1.13  [0.64; 1.99] | 1.33  [0.77; 2.29] |
| 3- to 24-month period | 0.88  [0.73; 1.05] | 0.98  [0.78; 1.23] | 1.06  [0.83; 1.35] | 1.03  [0.59; 1.80] | 1.27  [0.75; 2.17] |
| **Non-hip non-vertebral fracture (IRR [95% CI])** | | | | | |
| Baseline period | 1 | 1 | 1 | 1 | 1 |
| 3- to 12-month period | 0.91  [0.81; 1.02] | 1.09  [0.92; 1.31] | 1.05  [0.90; 1.22] | 1.40  [0.86; 2.27] | 1.81  [1.23; 2.68] |
| 3- to 18-month period | 0.90  [0.80; 1.01] | 1.16  [0.98; 1.37] | 1.02  [0.88; 1.17] | 1.39 0.87; 2.21] | 1.66  [1.13; 2.42] |
| 3- to 24-month period | 0.90  [0.81; 1.00] | 1.19  [1.01; 1.40] | 1.00  [0.87; 1.15] | 1.29  [0.81; 2.05] | 1.61  [1.11; 2.34] |

BP: bisphosphonates; CI: confidence interval; IRR: incidence rate ratio.

Supplemental Table 5. Incidence rate ratios by site, treatment and exposure period in as-treated analysis with a risk window applied after treatment discontinuation (sensitive analysis 2)

|  | **Denosumab N = 67,046** | **Oral BPs N = 52,914** | **Zoledronic acid N = 41,700** | **Raloxifene N = 11,660** | **Teriparatide N = 7,510** |
| --- | --- | --- | --- | --- | --- |
| **Vertebral fracture, IRR [95% CI])** | | | |  |  |
| Baseline period | 1 | 1 | 1 | 1 | 1 |
| 3- to 12-month period | 0.67  [0.54; 0.82] | 0.61  [0.45; 0.82] | 0.37  [0.30; 0.44] | 0.59  [0.19; 1.79] | 0.41  [0.29; 0.57] |
| 3- to 18-month period | 0.62  [0.51; 0.76] | 0.69  [0.53; 0.91] | 0.38  [0.31; 0.45] | 0.56  [0.20; 1.62] | 0.35  [0.25; 0.48] |
| 3- to 24-month period | 0.58  [0.48; 0.70] | 0.71  [0.54; 0.93] | 0.36  [0.30; 0.43] | 0.51  [0.18; 1.44] | 0.36  [0.26; 0.49] |
| **Hip fracture, IRR [95% CI])** | | | | | |
| Baseline period | 1 | 1 | 1 | 1 | 1 |
| 3- to 12-month period | 0.87  [0.71; 1.06] | 1.74  [1.26; 2.41] | 1.01  [0.81; 1.26] | 0.36  [0.05; 2.54 | 1.25  [0.72; 2.18] |
| 3- to 18-month period | 0.80  [0.66; 0.97] | 1.90  [1.39; 2.60] | 0.93  [0.75; 1.15] | 1.27  [0.28; 5.80] | 1.27  [0.74; 2.16] |
| 3- to 24-month period | 0.79  [0.65; 0.95] | 1.93  [1.42; 2.62] | 0.91  [0.74; 1.13] | 1.57  [0.36; 6.87] | 1.24  [0.73; 2.11] |
| **Wrist/forearm fracture IRR [95% CI])** | | | | | |
| Baseline period | 1 | 1 | 1 | 1 | 1 |
| 3- to 12-month period | 0.86  [0.70; 1.05] | 0.95  [0.74; 1.22] | 1.13  [0.87; 1.47] | 1.05  [0.57; 1.92] | 1.25  [0.70; 2.21] |
| 3- to 18-month period | 0.85  [0.70; 1.03] | 0.98  [0.77; 1.24] | 1.10   [0.85; 1.41 | 1.03  [0.57; 1.84] | 1.23  [0.70; 2.13] |
| 3- to 24-month period | 0.85  [0.71; 1.03] | 0.98  [0.78; 1.24] | 1.07  [0.83; 1.37] | 0.95  [0.53; 1.69] | 1.22  [0.70; 2.12] |
| **Non-hip non-vertebral fracture (IRR [95% CI])** | | | | | |
| Baseline period | 1 | 1 | 1 | 1 | 1 |
| 3- to 12-month period | 0.87  [0.77; 0.99] | 1.09  [0.91; 1.30] | 1.04  [0.90; 1.21] | 1.41  [0.86; 2.31] | 1.80  [1.21; 2.66 |
| 3- to 18-month period | 0.85  [0.75; 0.96] | 1.16  [0.98; 1.38] | 1.00  [0.86; 1.16] | 1.36  [0.84; 2.20] | 1.60  [1.09; 2.35] |
| 3- to 24-month period | 0.84  [0.74; 0.94] | 1.19  [1.01; 1.40] | 0.98  [0.84; 1.13] | 1.25  [0.78; 2.00 | 1.59  [1.09; 2.33] |

BP: bisphosphonates; CI: confidence interval; IRR: incidence rate ratio.
* 365-day risk window for oral bisphosphonates group and 30-day risk window for others

Supplemental Figure 1. On-treatment incidence rate ratios for fractures by site and by treatment, stratified by age

Supplemental Figure 2. On-treatment incidence rate ratios for fractures by site and by treatment, stratified by history of fracture

Supplemental Figure 3. On-treatment incidence rate ratios for fractures by site and by treatment, stratified by glucocorticoid dispensing

Supplemental Figure 4. On-treatment incidence rate ratios for fractures by site and by treatment, stratified by history of osteoporosis medication

Supplemental Figure 5. On-treatment incidence rate ratios for fractures by site and by treatment, stratified by history of dementia

Supplemental Figure 6. On-treatment incidence rate ratios for fractures by site and by treatment, stratified by history of nervous system disorders (excluding Parkinson disease)
